# Continuous Synthesis of Uniformly Dispersed Mesoporous SBA-15 Supported Silver Nanoparticles in a Coiled Flow Inverter Reactor

**DOI:** 10.3389/fchem.2021.747105

**Published:** 2021-09-22

**Authors:** Hai Zhu, Ke-Jun Wu, Chao-Hong He

**Affiliations:** ^1^ Zhejiang Provincial Key Laboratory of Advanced Chemical Engineering Manufacture Technology, College of Chemical and Biological Engineering, Zhejiang University, Hangzhou, China; ^2^ Institute of Zhejiang University-Quzhou, Quzhou, China; ^3^ School of Chemical and Process Engineering, University of Leeds, Leeds, United Kingdom

**Keywords:** Ag/SBA-15, mesoporous-SBA, uniformly dispersed, structure of support, continuous flow reactor

## Abstract

Mesoporous silica supported nanocatalysts have shown great potential in industrial processes due to their unique properties, such as high surface area, large pore volume, good chemomechanical stability and so on. Controllable and tunable synthesis of supported nanocatalysts is a crucial problem. Continuous synthesis of supported nanoparticles has been reported to get uniformly dispersed nanomaterials. Here, a method for continuous synthesis of uniformly dispersed mesoporous SBA-15 supported silver nanoparticles in a coiled flow inverter (CFI) microreactor is described. Compared to Ag/SBA-15 synthesized in the conventional batch reactor and Ag synthesized in continuous flow, mesoporous silica nanocatalysts synthesized in continuous flow are found to have smaller average size (7–11 nm) and narrower size distribution. The addition of capping agents can effectively change the characteristic of catalysts. Moreover, two kinds of support with different surface area and pore size have been added into the continuous synthesis. This method can provide further understandings for the synthesis of uniformly dispersed supported nanocatalysts in continuous flow, especially for mesoporous nanomaterials, which provides the possibilities of large-scale yield process of supported nanocatalysts in industry.

## Introduction

Supported nanocatalysts have been widely used in the chemical and pharmaceutical industries (Tu et al., 2017; Yang et al., 2019). The support can greatly improve nanoparticles’ dispersion and inhibit aggregation, extending the catalyst service life and enhancing the catalytic activity (Long et al., 2013; Zhang et al., 2020). To date, various materials have been used as the support, such as metal-organic frameworks (MOF) (Llabrés i Xamena et al., 2008), zeolites (Song et al., 2021), carbon nanotubes (CNT) (Serp and Castillejos, 2010) and mesoporous silicas (Mohammadi Ziarani et al., 2018). SBA-15, a hydride material of mesoporous silicas, has been developed in heterogeneous catalysis due to its uniform pore size, large surface area and high thermal and chemical stability (Singh et al., 2018; Tieuli et al., 2019; Wu et al., 2021). SBA-15 supported metal nanocatalysts, such Pt/SBA-15 (Lai et al., 2014), Au/SBA-15 (Soni et al., 2018), Ag/SBA-15 (Qu et al., 2012), have shown a great catalytic activity for catalytic oxidation of volatile organic compounds (VOCs). Among these metal nanocatalysts, silver nanoparticles have been widely investigated because of their high electrical conductivity, pyroconductivity, and chemical stability (Yonezawa et al., 2000; Kawai et al., 2012; Shen et al., 2014).

In the past few decades, various methods, such as precipitation (Arshad et al., 2019; Rahdar et al., 2019), sol-gel (Delir Kheyrollahi Nezhad et al., 2016) and high-temperature pyrolysis (Chen et al., 2020), have been developed to synthesize supported nanocatalysts. These methods were developed mainly in a batch reactor, which may lead to polydispersity of nanoparticles and batch-to-batch irreproducibility (Marre and Jensen, 2010). Since the catalytic performance of supported nanocatalysts is highly related to the size, shape, composition, and etc. (Mäki-Arvela and Murzin, 2013), controlled and tunable synthesis of supported nanomaterials has started gaining more attention.

Many strategies, such as microwave heating (Fuku et al., 2013; Verma et al., 2015) and functionalization of support (Saikia et al., 2016), have been proposed to control the particle size and size distribution based on batch process. However, they may still suffer from batch to batch variations, poor mixing and poor heat and mass transfer, especially for large-scale production. Micro- and milli-fluidic reactors are often employed to synthesize nanoparticles owing to their unique characteristics, e.g., high heat and mass transfer, operational safety and enhanced scalability (Jensen, 2017; Besenhard et al., 2020; De Risi et al., 2020). Size-controlled and highly dispersed nanoparticles have been synthesized in various types of microreactors, such as jet-mixing reactor (Ranadive et al., 2019), droplet-based microreactor (Kim et al., 2020) and coiled flow inverter reactor (Wu et al., 2017). However, the heterogeneous synthesis of supported nanoparticles in microreactors has rarely been mentioned. Cattaneo et al. reported the synthesis of highly uniform and composition controlled TiO_2_-supported bimetallic Au-Pd nanoparticles in continuous flow (Cattaneo et al., 2019). The as-synthesized nanoparticles showed a smaller mean particle size (ca. 2 nm) and improved size distribution compared with the conventional sol-immobilization method. On the other hand, mesoporous materials, which have a unique pore structure, have been shown to enhance the dispersion and catalytic activity of the supported nanocatalysts (Liang et al., 2017; Liu et al., 2017; Thennila et al., 2020). However, continuous synthesis of mesoporous silica nanocatalysts has gained little attention, and the effect of their special surface area on the nucleation and growth in continuous synthesis is not yet discussed.

Actually, several types of microreactors have been involved in nanoparticles’ synthesis as mentioned before. Conventional capillary or tube microfluidic reactors have attracted attention due to their unique characteristics such as simplicity, robust materials and high temperature adaptability (Zhao et al., 2011). However, the polydispersity in the nanoparticle sizes can not be avoided because of broaden velocity and residence time distributions as the laminar flow (Wagner and Tshikhudo, 2008; Sebastian Cabeza et al., 2012). Segment microreactors can form bubbles or liquid droplets, which can enhance mixing and decrease axial dispersion by dividing the fluids into segments. Complexity of the system and the separation of the resulting nanoparticles can cause many problems, especially for droplet-based microreactors (Teh et al., 2008). Coiled flow inverter reactor can enhance the axial mixing and narrow the time distribution due to the secondary flow, leading to the narrower size distribution. We have demonstrated continuous synthesis of controlled and tunable nanoparticles in a CFIR without any capping agents (Wu et al., 2017; Wu et al., 2018; Wu and Torrente-Murciano, 2018; Bailey et al., 2021).

Therefore, in this work, we present a method for size-controllable synthesis of Ag/SBA-15 nanoparticles in a coiled flow inverter reactor (CFIR) via an impregnation-reduction method. The process was investigated by changing several operational parameters, such as flow rate and initial concentration. Then, polyvinyl pyrrolidone (PVP) was added into the solution to further investigate the effect of capping agents on the nucleation and growth of the nanoparticles. Moreover, the effect of the supports with various structure parameters on the nanoparticles’ size and size distribution during the synthesis is explored and discussed. Finally, the relationship between nanoparticle size, loading content and activity is illustrated here via the evaluation of the catalytic performance of the reduction of 4-nitrophenol.

## Expection Section

### Chemicals

Silver nitrate (AgNO_3_), sodium borohydride (NaBH_4_), 4-nitrophenol and polyvinyl pyrrolidone (PVP) are analytical grade and purchased from Sinopharm Chemical Reagent Co., Ltd, China. Two kinds of SBA-15 were purchased from JCNANO Tech Co., Ltd, China. The detailed structure information is shown in Table 1. All the chemical reagents were prepared with deionized water.

**TABLE 1:** Structure information of the two types of SBA-15.

Sample	Specific surface area (m^2^/g)	Pore diameter (nm)
SBA-15	870	9.5
SBA-15-4.2	759	5.6

### Synthesis of Silver Nanoparticles

The nanocatalysts were synthesized through the impregnation-reduction method. The precursors were reduced by sodium borohydride. The nucleation process occurred in several milliseconds and the reduced nuclei grew and formed nanoparticles. Ultrasonic was introduced when mixing the precursors to increase the deposition amounts.

### Batch Synthesis of Supported Silver Nanoparticles

Batch synthesis was conducted in a 250 ml flask. First, 0.2 mM silver nitrate solution was mixed with SBA-15 and the solution was sonicated for 2 h. Then, 10 mM NaBH_4_ was added to the precursor solution. The mixture was stirred at room temperature for 35 s. The products were washed with deionized water and separated by centrifugation several times. Finally, the Ag/SBA-15 was dried at 90°C overnight.

### Flow Synthesis of Silver Nanoparticles

**FIGURE 1:**
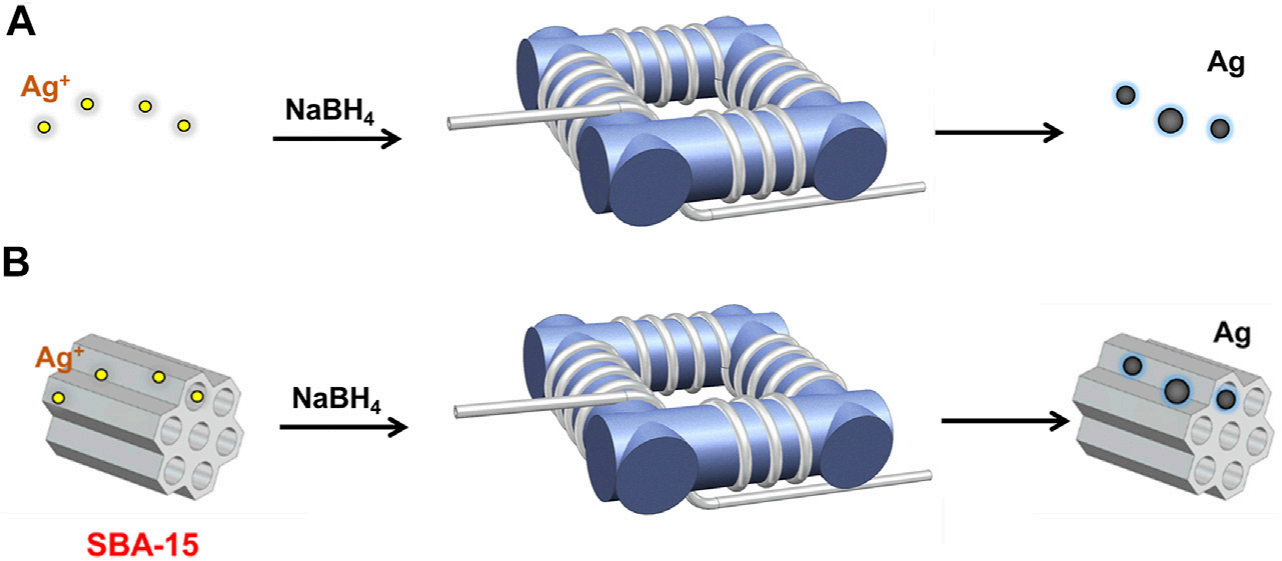
Schematic diagram of the experimental setup for **(A)** silver nanoparticle synthesis, and **(B)** supported silver nanocatalysts synthesis.

As illustrated in Figure 1A, the silver nanoparticles were synthesized in a coiled flow inverter reactor (CFIR), which consists of perfluoroalkoxy tubing (PFA, IDEX Health & Science LLC) with a 0.03 inch inner diameter and 1/16 inch outer diameter and a 3D-printed support framework. In this work, the framework was printed using FormLabs 2 stereolithography printer from FormLabs to get precise geometric parameters, i.e., helix diameter (1 cm), pitch distance (0.5 cm), and channel length. In the process, 0.2 mM silver nitrate solution and 10 mM sodium borohydride solution were introduced into the CFIR using two syringe pumps (LSP01-3A, Longer Pump). The flow rates of NaBH_4_ and AgNO_3_ were 0.4 and 2 ml/min, respectively. Then, the products were collected in a centrifuge tube immersed in an ice-water bath.

### Flow Synthesis of Supported Silver Nanoparticles

The supported silver nanoparticles were synthesized in a CFIR, the same structure as mentioned before (as shown in Figure 1B). Before the reaction, SBA-15 was added into silver nitrate solution followed by sonication for 2 h. In a typical process, the prepared precursor mixture and sodium borohydride solution were introduced into the CFIR using two syringe pumps (LSP01-3A, Longer Pump) with a fixed flow rate ratio and concentration ratio, i.e., $Q_{AgNO_{3}}:Q_{NaBH_{4}}\;=\;5:1,\;C_{AgNO_{3}}:C_{NaBH_{4}}\;=\;1:50.$ Then, the products were collected in a centrifuge tube immersed in an ice-water bath. Finally, the collected products were washed with deionized water and separated by centrifugation several times. The obtained Ag/SBA-15 were dried at 90°C overnight.

### Characterization

Powder X-ray diffraction (XRD) patterns were collected using a PANAlytical X'pert3 Powder X-ray diffractometer (PANAlytical, Holland). Ultraviolet-visible (UV-Vis) spectroscopy measurements were performed on a 756 UV-vis spectrophotometer in the 300–700 nm wavelength range with a resolution of 1 nm. Transmission Electron Microscopy (TEM) was carried out with HT-7700 (Tokyo, Japan) operating at 100 kV. The metal content of obtained catalysts was analyzed by a Varian 730-ES type inductively coupled plasma atomic emission spectroscopy (ICP-AES) instrument (Varian, America). The Brunauer-Emmett-Teller (BET) specific surface areas and pore volume of the support were characterized by nitrogen adsorption–desorption (AUTOSORB-1-C, America).

### Evaluation of Catalytic Performance of the Catalyst

The reduction of 4-nitrophenol with NaBH_4_ was conducted to evaluate the catalytic performance. Before the reaction, the obtained dry catalysts were dispersed in deionized water, and the silver concentrations was just controlled at 0.2 mM. 1 ml of 1.5 × 10^−4^ M 4-nitrophenol aqueous solution, 1 ml of 10^−1^ M sodium borohydride aqueous solution, and 100 µl of as-prepared supported nanoparticles were added into a quartz cuvette. After adding as-prepared nanoparticles, the time-dependent UV-Vis absorbance spectra were recorded immediately to analyze the reaction rate. The background correction was done with deionized water only.

## Results and Discussion

### Batch and Flow Synthesis of Supported Nanoparticles

**FIGURE 2:**
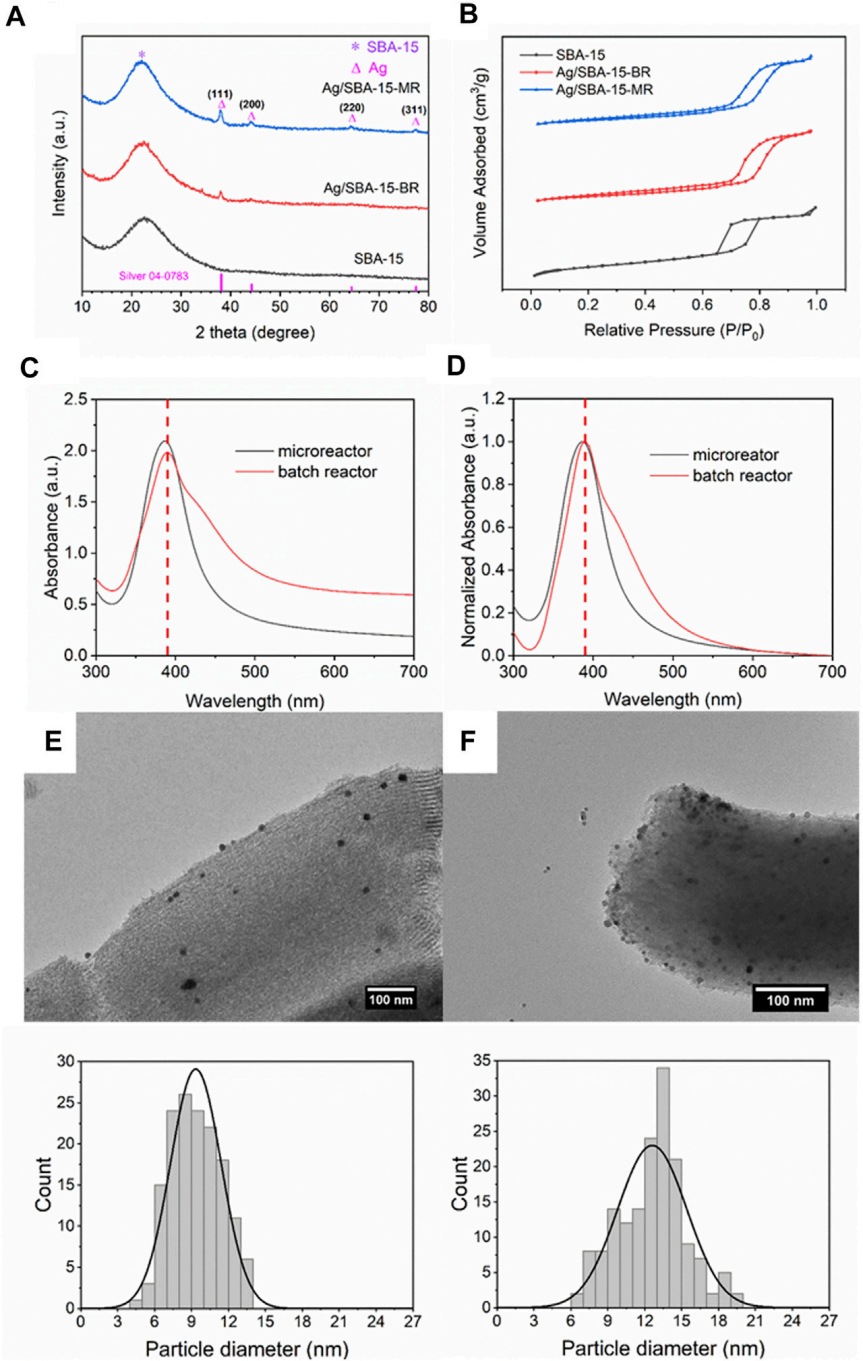
**(A)** XRD patterns, **(B)** N_2_ adsorption-desorption isotherms, **(C)** UV-Vis absorbance spectra and **(D)** normalized absorbance spectra of Ag/SBA-15 synthesized in a coiled flow inverter microreactor and a batch reactor. TEM images and size distribution of silver nanoparticles dispersed on SBA-15 synthesized in **(E)** a microreactor (9.35 ± 2.06 nm), and **(F)** a batch reactor (12.61 ± 2.81 nm).

The residence time distribution was different for the batch reactors and continuous flow reactors, i.e., the nucleation rate and growth rate for nanoparticles were different. Then, in this process, Ag/SBA-15 was synthesized in a batch reactor and a CFIR, with the same concentration and residence time. The crystalline phase of Ag/SBA-15 synthesized at different reactors was investigated by XRD, as shown in Figure 2A. The broad peak at a 2*θ* value of 10–30° is ascribed to the amorphous pore wall structure of the SBA-15. When the silver was deposited on the SBA-15, the sharp diffraction peaks at 38°, 44°, 64° and 77°, which respectively correspond to the (111), (200), (220) and (311) planes of cubic phase of Ag (JCPDS card No. 04-0783), could conform the presence of Ag NPs at SBA-15 surface (Kayal et al., 2019). The mesoporous structure of the support is stable during the synthesis process, even in a microreactor. The peak for Ag/SBA-15 synthesized in a microreactor is stronger than that synthesized in a batch reactor, due to the increase of actual loading content.

N_2_ adsorption-desorption isotherms for SBA-15 and Ag/SBA-15 synthesized in batch reactor and continuous flow are found to be type-IV isotherm with H1 hysteresis loop as indicated in Figure 2B, which is the characteristic of typical mesoporous material (Naik et al., 2011). The Brunauer–Emmett–Teller specific surface area for SBA-15 is calculated to be 870 m^2^/g and the pore diameter is about 9.5 nm. The specific surface area for Ag/SBA-15 synthesized in batch reactor and a microreactor is respectively 411 and 400 m^2^/g. The decrease of the specific surface area was attributed to the silver successfully incorporated into the mesopores of SBA-15. The pore diameters are 8.2 and 8.1 nm for Ag/SBA-15-BR and Ag/SBA-15-MR. The increase of silver loading content induce the slight decrease of specific surface area and pore diameter for the nanomaterials synthesized in continuous flow.

As Figures 2C,D show, nanocatalysts synthesized in a batch reactor exhibit a broader absorbance due to the larger size of nanoparticles. A red shift of the absorbance peak was observed, which also indicated the average size of nanocatalysts prepared using the batch reactor is relatively larger. Compared to the catalyst synthesized in a CFIR, the full width at half maximum (FWHM) value of nanocatalyst synthesized in a batch reactor increases from 66 to 86 nm. That is because in the batch reactor, the reagents were added into the solution immediately, and reductants, and precursors were not mixed sufficiently due to the poor macromixing in batch reactors (Ranadive et al., 2019). Residence time distribution was broad and agglomeration was severe in the batch reactor. Thus, larger nanoparticles with broader size distribution were obtained. For the synthesis in the coiled flow inverted reactor, high heat and mass transfer narrow the residence time distribution, and secondary flow can further enhance this process.

In order to further confirm the results obtained by UV-Vis, the TEM measurement was conducted to get accurate particles size and size distribution, as shown in Figures 2E,F. For the silver nanoparticles synthesized in the batch reactor, the average particle size was 12.61 nm, larger than that synthesized in continuous flow. Also, narrower size distribution in the CFIR was obtained owing to the enhanced mass transfer.

### Flow Synthesis of Nanoparticles and Supported Nanoparticles

**FIGURE 3:**
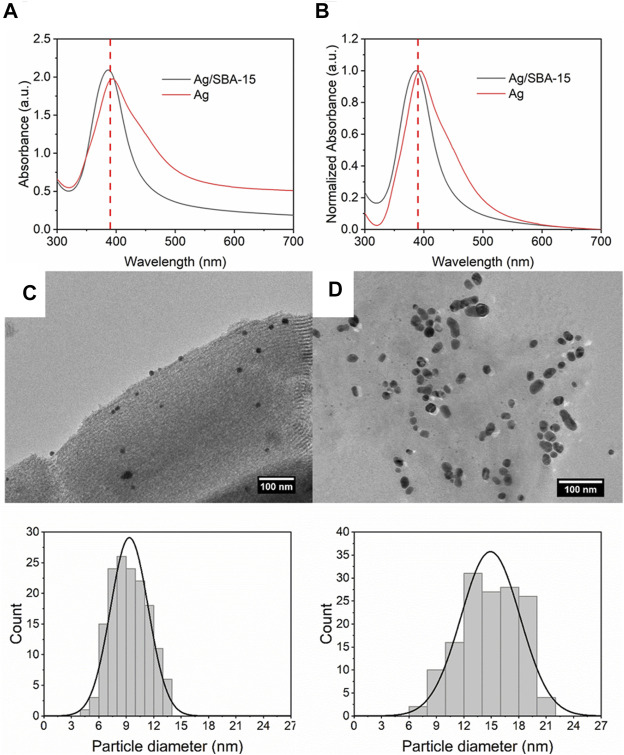
**(A)** UV-vis absorbance spectra and **(B)** normalized absorbance spectra for Ag/SBA-15 and Ag nanoparticle synthesized in a coiled flow inverter microreactor. TEM images and size distribution of silver nanoparticles synthesized **(C)** with SBA-15 support (9.35 ± 2.06 nm), and **(D)** without SBA-15 support (14.91 ± 3.22 nm).

In order to investigate the effect of support on the synthesis of nanoparticles, the nanoparticles without SBA-15 were prepared. The initial concentration of silver precursor was 0.2 mM, and the flow rate of the precursor was 2 ml/min. For the supported nanocatalysts, the loading content was 5 wt%, and the initial concentration and flow rate were kept the same. As shown in Figures 3A,B, it is interesting to find a red shift (ca. 5 nm), which indicates that the average size of the nanoparticles is larger for the nanoparticles without support. Also, the FWHM value increases from 66 to 89 nm. Therefore, the size distribution was broader for Ag nanoparticles.

Accurate particle size was obtained by the TEM measurement. Figure 3C shows that the average size of Ag dispersed on SBA-15 is 9.35 ± 2.06 nm, smaller and narrower than Ag (Figure 3D), which is consistent with the UV results. The differences of results were induced by the introduction of SBA-15. When SBA-15 is introduced into the system as support, as a result, some precursor solution enters into the pore, then it diffuses and adsorbs on the pore (Mäki-Arvela and Murzin, 2013). When adding the reagents into the solution, the reagents diffuse into the pore and react with the precursor (Kim et al., 2009). Every pore can be treated as a small reactor, i.e., the concentrations of the reagents in the pore are higher. So, the fast nucleation process leads to a smaller size for the mesoporous silica nanocatalysts. In addition, in the growth process, the good dispersion of the support can reduce agglomeration, which can help synthesize nanoparticles with narrower size distribution. Above all, it can explain the results obtained by UV-Vis and TEM.

### Effect of Synthesis Parameters in Continuous Flow

#### Effect of Flow Rate

**FIGURE 4:**
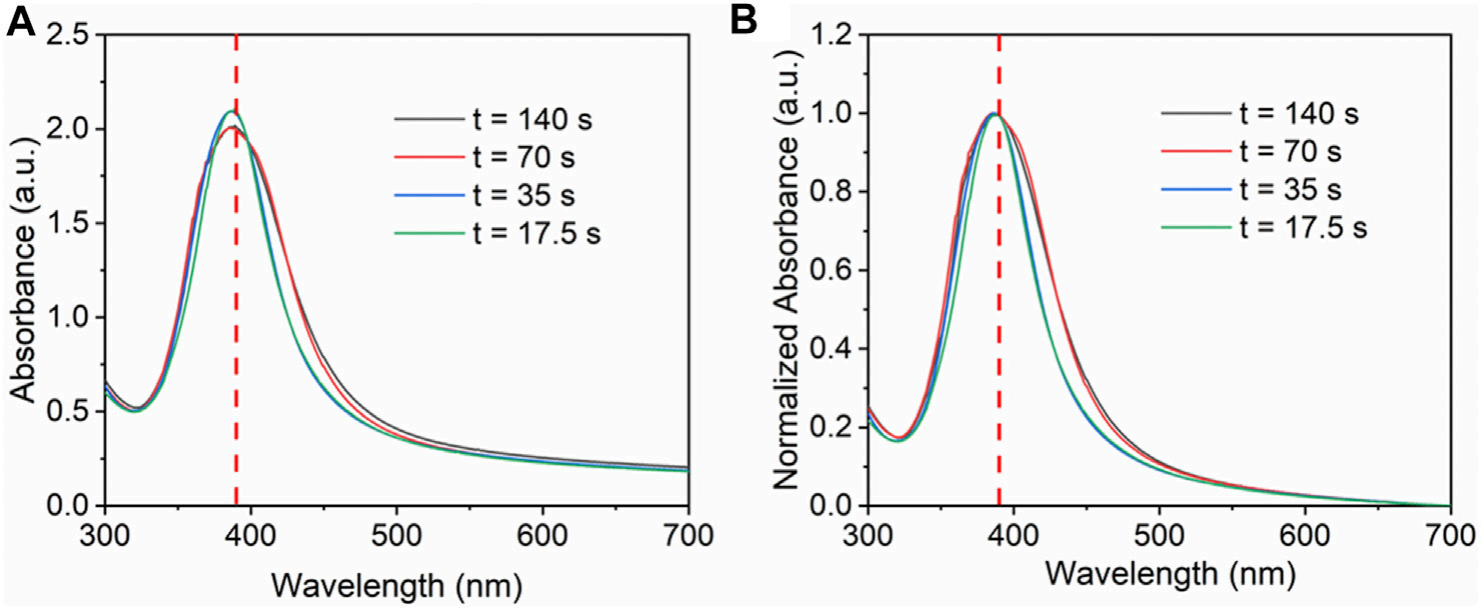
**(A)** UV-vis absorbance spectra and **(B)** normalized absorbance spectra for Ag/SBA-15 synthesized at different flow rates.

In the microreactors, change in flow rate not only changes the residence time, but also changes the mixing and turbulence, thus influencing the nucleation and growth of nanoparticles (Dong et al., 2020). In this process, the flow rate of precursor mixture varies from 0.5 ml/min (*t* = 140 s) to 4 ml/min (*t* = 17.5 s), while other conditions such as the initial concentration of silver precursor (0.2 mM), the loading content (5 wt%) and the flow rate ratio of AgNO_3_ and NaBH_4_ (5:1), were kept constant. SBA-15 with 9.5 nm pore diameter was added to the solution as support. The UV-Vis spectra in Figure 4 show that there is no significant change for the absorbance peak when increasing the flow rate. No red shift or blue shift is observed. The FWHM value changes from 62 to 79 nm. Therefore, when changing the flow rate, size and size distribution was similar.

**FIGURE 5:**
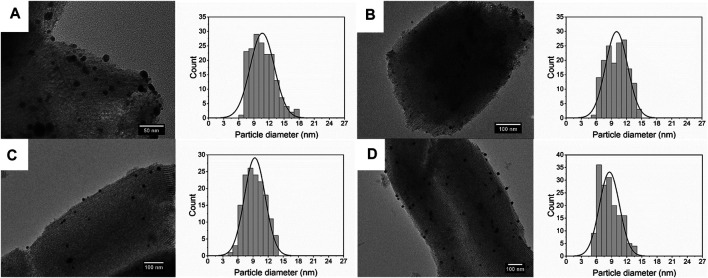
TEM images and size distribution of silver nanoparticles dispersed on SBA-15 synthesized at residence time of **(A)** *t* = 140 s (10.66 ± 2.39 nm), **(B)** *t* = 70 s (10.02 ± 2.19 nm), **(C)** *t* = 35 s (9.35 ± 2.06 nm), and **(D)** *t* = 17.5 s (8.58 ± 1.98 nm).

Figure 5 shows the size of Ag deposited on SBA-15 synthesized at different residence time. The size of Ag nanoparticles decreases from 10.66 ± 2.39 to 8.58 ± 1.98 nm, along with the increase of flow rate. When the flow rate increased, the turbulence was enhanced, thus the nucleation rate increased, leading to smaller size of nanoparticles. On the other hand, residence time increases with the decrease in flow rate, meaning there was more time for agglomeration, which generated nanocatalysts with broad size distribution.

For the supported nanocatalysts, the loading content is a vital factor for the catalytic activity. An increase of active sites will facilitate catalytic activity. The actual loading contents for nanocatalysts synthesized at different flow rates are shown in Table 2. When increasing the residence time, the actual loading content increases from 2.84 to 3.27 wt%. The actual loading ratio did not reach 100% even when the residence time was 140 s. Also, in these synthesis process, all the obtained solution after centrifugation separation were yellow, which indicated that there were dissociative silver in the solution. According to that, it can be inferred that, after pre-treatment, some silver ions were deposited on the support, however, some were suspending in the solution. These dissociative silver ions were reduced to form silver nanoparticles in the solution and randomly collided with dispersed SBA-15. Thus, some dissociative nanoparticles were deposited on the support. At a fast flow rate, the residence time was less, and the turbulence was violent. As a result, fewer dissociative nanoparticles were deposited on the support (Zanella et al., 2005).

**TABLE 2:** Actual loading content of supported nanocatalysts measured by ICP-AES.

Sample number	Concentration of silver precursor (mM)	Residence time (s)	Content of Ag (wt%)
1	0.2	17.5	2.84
2	0.2	35	2.91
3	0.2	70	3.13
4	0.2	140	3.27

### Effect of Initial Concentration

**FIGURE 6:**
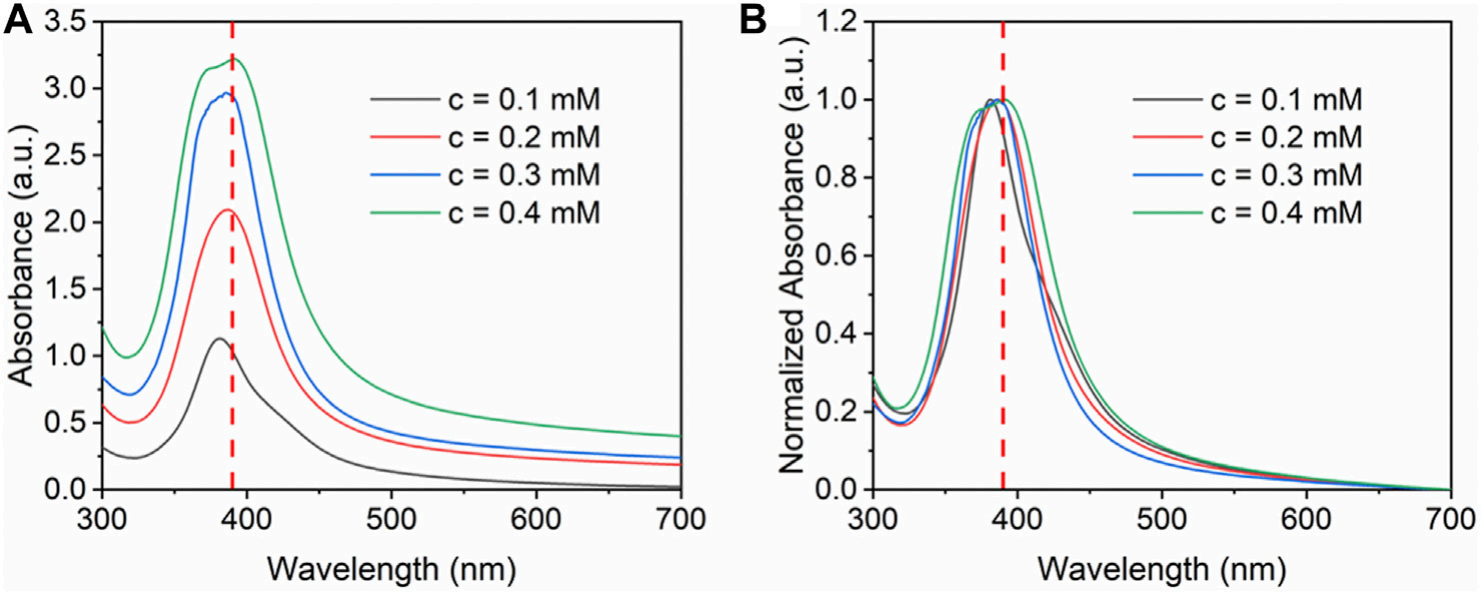
**(A)** UV-vis absorbance spectra and **(B)** normalized absorbance spectra for Ag/SBA-15 synthesized at different concentration.

The effect of silver precursor concentration was investigated by altering the initial concentration from 0.1 to 0.4 mM. The flow rate of the precursor was kept at 2 ml/min, and the loading content was kept as 5 wt%. The concentration ratio of NaBH_4_ and Ag precursors was controlled at 50:1. Furthermore, the flow rate of NaBH_4_ was 0.4 ml/min. It can be seen from Figure 6 that when increasing the initial concentration, higher absorbance was obtained due to the higher concentration of silver nanoparticles. Due to the agglomeration at high concentration, the FWHM value increases gradually from 63 to 83 nm with the increase of concentration. At high concentration, the absorbance peak has a slight red shift which indicates larger nanoparticles synthesized.

**FIGURE 7:**
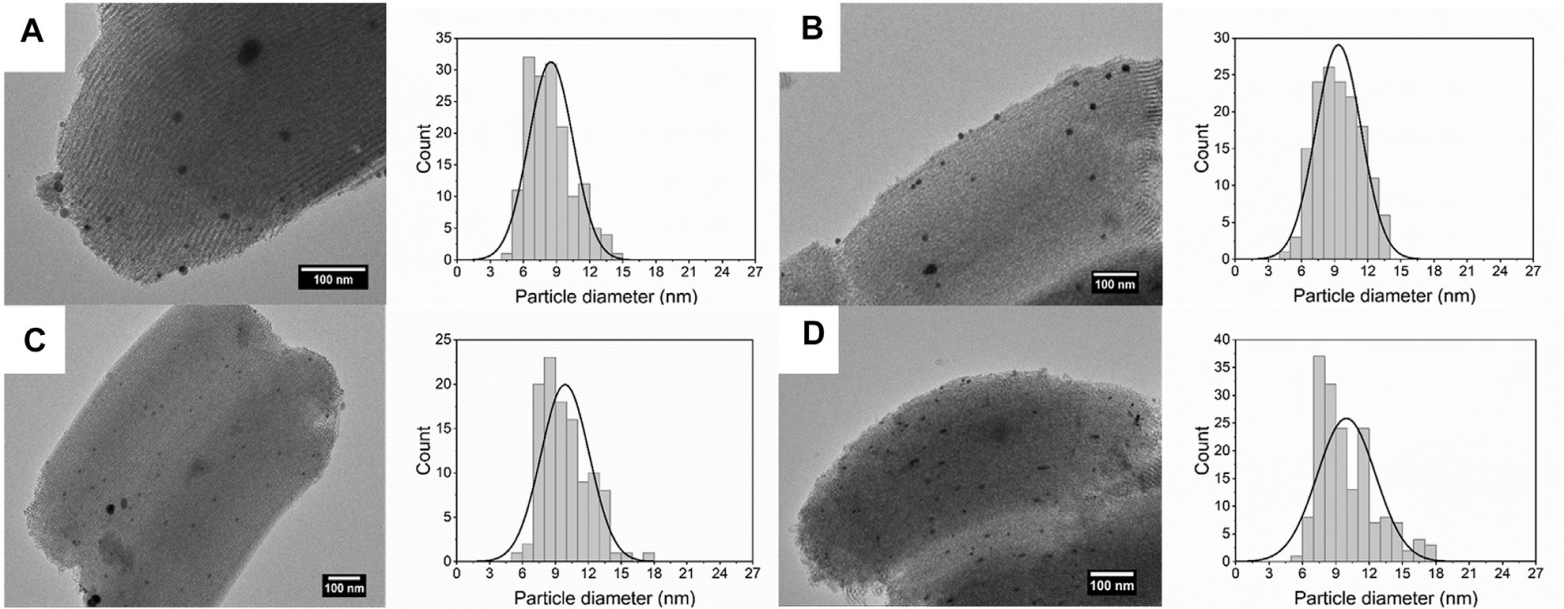
TEM images and size distribution of silver nanoparticles dispersed on SBA-15 synthesized at silver concentration of **(A)** 0.1 mM (8.48 ± 2.00 nm), **(B)** 0.2 mM (9.35 ± 2.06 nm), **(C)** 0.3 mM (9.88 ± 2.20 nm), and **(D)** 0.4 mM (10.33 ± 2.63 nm).

The average size and size distribution were further determined using the TEM measurement and the results are shown in Figure 7. With the increase in concentration, the nucleation and growth process are facilitated, and the nanoparticles agglomerate more possibly at high concentration, therefore, the average size of silver nanoparticles increases from 8.48 ± 2.00 to 10.33 ± 2.63 nm.

Actual loading contents were depicted in Table 3. Theoretically, the actual loading content of silver should be same when changing the initial concentration of silver because calculated loading content and flow rate were kept constant, meaning residence time and turbulence had no remarkable change. However, as the concentration increases, the actual loading contents decrease slightly, from 3.08 to 2.79 wt%. Taking the amount of support into consideration, the phenomenon can have a reasonable explanation. As the loading content was kept same, more supports were introduced into the reactor when initial concentration increased. The suspending solids were deposited on the channel and clogged in the tube (Marre and Jensen, 2010; Gao et al., 2020). Thus, less silver nanoparticles were deposited on the support at the outlet of the reactor.

**TABLE 3:** Actual loading content of supported nanocatalysts measured by ICP-AES.

Sample number	Concentration of silver precursor (mM)	Residence time (s)	Content of Ag (wt%)
1	0.1	35	3.08
2	0.2	35	2.91
3	0.3	35	2.85
4	0.4	35	2.79

### Effect of Capping Agent

**FIGURE 8:**
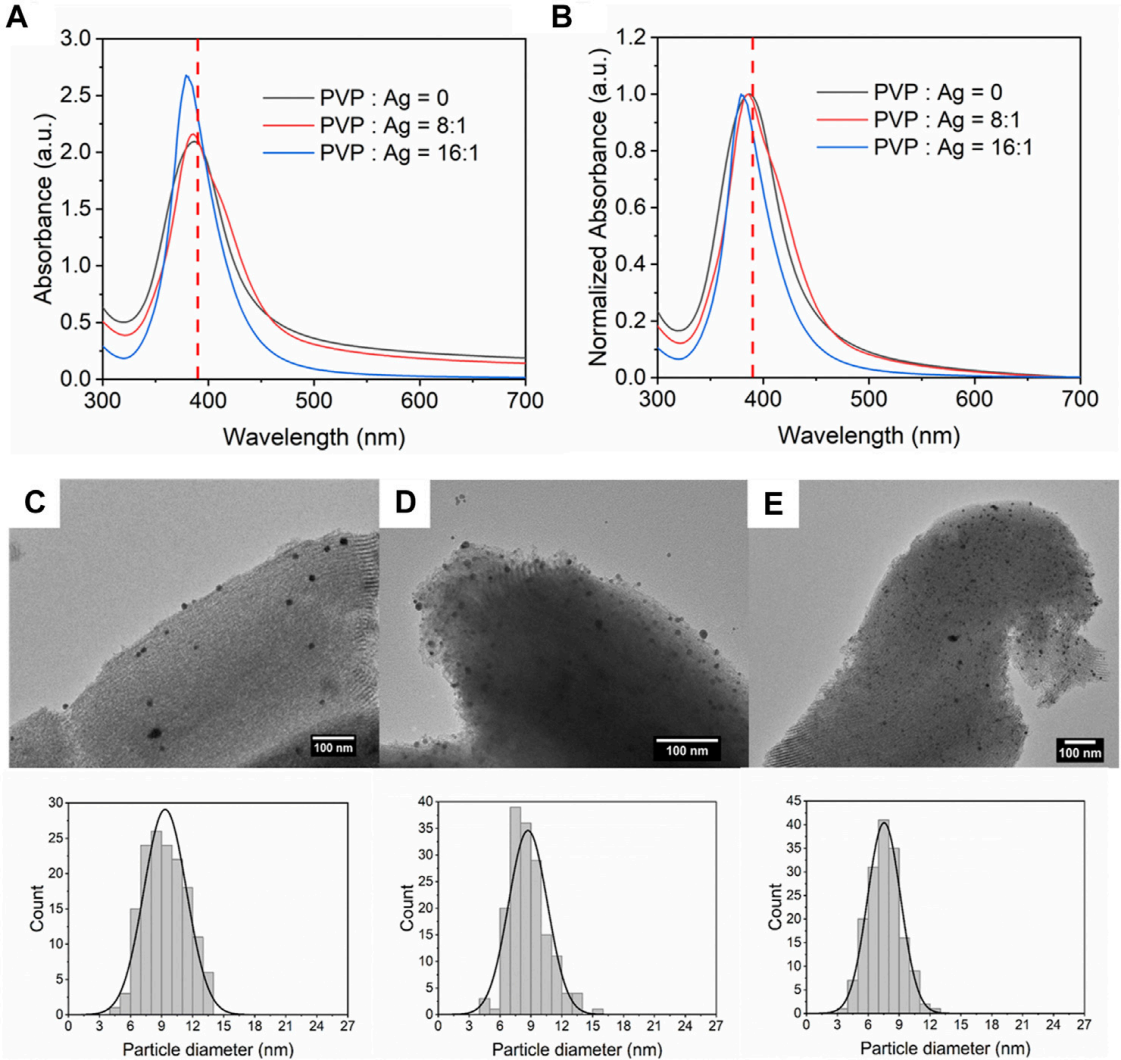
**(A)** UV-Vis absorbance spectra and **(B)** normalized absorbance spectra for Ag/SBA-15 synthesized at different concentration ratio of PVP and silver nitrate. TEM images and size distribution of silver nanoparticles dispersed on SBA-15 synthesized at PVP and AgNO_3_ concentration ratio of **(C)** 0:1 (9.35 ± 2.06 nm), **(D)** 8:1 (8.27 ± 1.88 nm), and **(E)** 16:1 (7.63 ± 1.77 nm).

Steric organic capping agents were often involved in the synthesis of size-controlled nanoparticles as a surface stabilizer and nanoparticle dispersant (Singh et al., 2018). Depending on the specific conditions, polyvinyl pyrrolidone (PVP) can react with the precursors as a reducing agent (Koczkur et al., 2015). Therefore, PVP was mixed with NaBH_4_ before being injected into CFIR to avoid reacting with the silver nitrate. Two different concentrations of PVP were introduced into the solution, $C_{PVP}\;:\;C_{Ag}\;=\;8:1\;\text{a}\text{n}\text{d}\;16:1$. The initial concentration of silver was kept constant at 0.2 mM. Other conditions were just kept the same as mentioned before. After adding PVP into the reaction, the UV-Vis spectra become narrower, as depicted in Figures 8A,B. It is interesting to find that the increase in concentration of PVP leads to sharper and higher absorbance peak due to the high dispersion induced by PVP. The FWHM value decreases from 66 to 46 nm. For the higher PVP concentration, the highest absorbance peak has a blue shift because of the smaller nanoparticle size.

Figures 8C–E show the accurate size and size distribution of Ag deposited on SBA-15 synthesized with different PVP concentrations. The average size of Ag dispersed on SBA-15 without PVP added is 9.35 ± 2.06 nm. When PVP was added into the solution, PVP would be coated at the surface of silver nanoparticles and serve as a surface stabilizer (Koczkur et al., 2015). Thus, the growth and agglomeration would be prevented. Therefore, silver nanoparticles deposited on SBA-15 was small and had narrow size distribution (8.27 ± 1.88 nm). With further increase in PVP concentration, more PVP were coated on the silver nanoparticles resulting in a smaller size and narrower size distribution (7.63 ± 1.77 nm).

According to the ICP-AES results, there is no significant difference for the actual loading contents with different concentrations of PVP, as demonstrated in Table 4. It indicates that the capping agents have no remarkable influence on the nanoparticles loading, but mainly influencing the size and size distribution of the synthesized nanoparticles.

**TABLE 4:** Actual loading content of supported nanocatalysts measured by ICP-AES.

Sample number	Concentration of silver precursor (mM)	Concentration ratio (PVP: NaBH4)	Content of Ag (wt%)
1	0.2	0 : 1	2.91
2	0.2	8 : 1	2.93
3	0.2	16 : 1	2.88

### Effect of Surface Area of the Support

**FIGURE 9:**
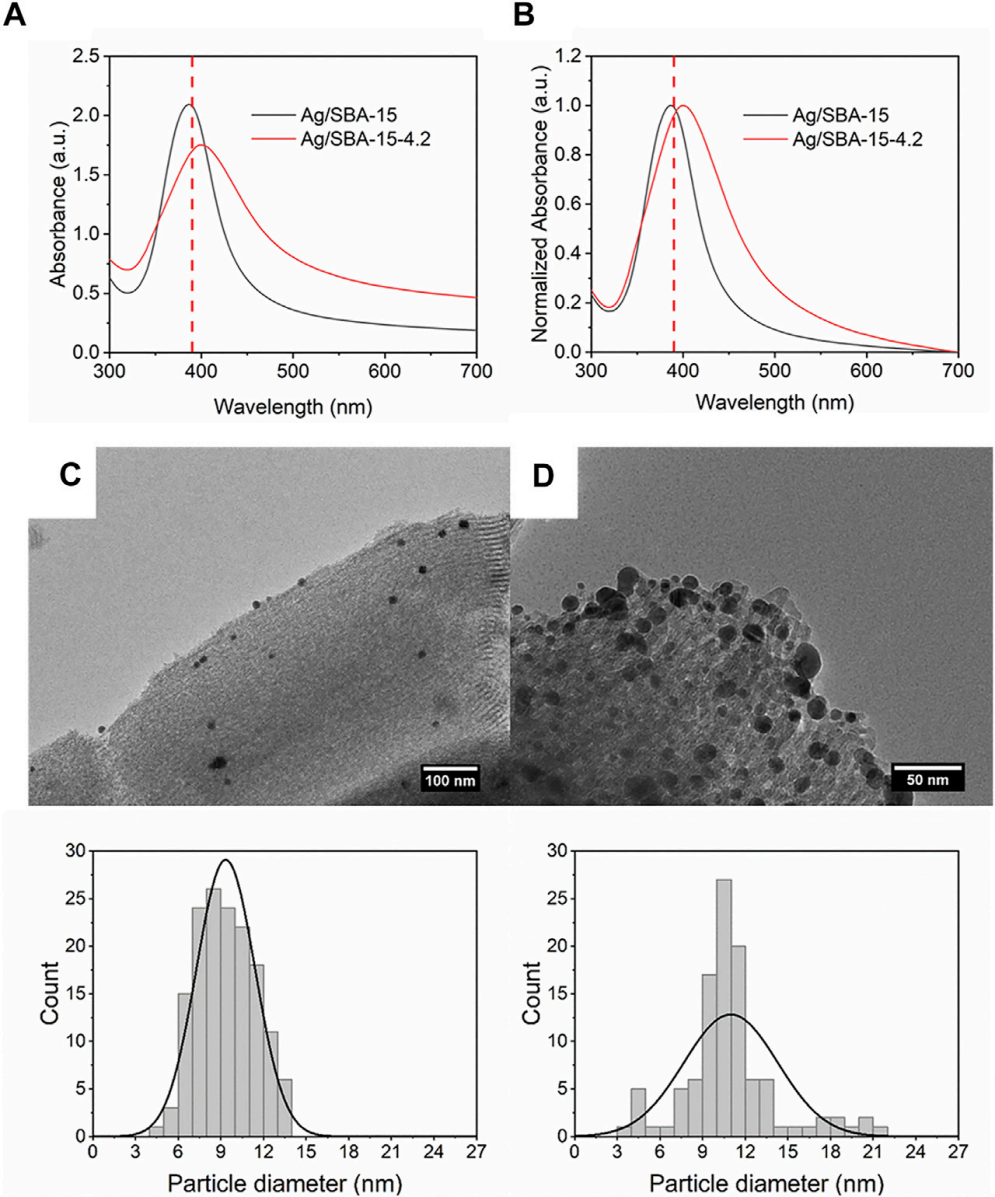
**(A)** UV-Vis absorbance spectra and **(B)** normalized absorbance spectra for Ag/SBA-15 and Ag/SBA-15-4.2. TEM images and size distribution of silver nanoparticles dispersed on SBA-15 and SBA-15-4.2 **(C)** Ag/SBA-15 (9.35 ± 2.06 nm), and **(D)** Ag/SBA-15-4.2 (10.98 ± 3.30 nm).

Characteristics of support, such as pore size and surface area, can remarkably affect the dispersion and efficiency of the metal precipitation, especially for the mesoporous materials (Miller et al., 2004). In this part, two types of SBA-15 with different surface areas were introduced into the solution. The concentration of silver precursor was 0.2 mM, and the flow rate was 2 ml/min. As shown in Figure 9, Ag/SBA-15-4.2 exhibits a lower and broader absorbance as a result of broad size distribution. FWHM value increases from 66 to 106 nm. A significant red shift of about 10 nm is observed for Ag/SBA-15-4.2, which indicates that the size of the synthesized Ag/SBA-15-4.2 is larger.

As is known, for mesoporous supports, a large specific surface area can serve as the contact area for the reaction. A larger surface area can facilitate the nucleation resulting in the small size of nanoparticles. In addition, a large surface area can disperse the nanoparticles and inhibit the agglomeration (Sankar et al., 2020). So that, particle size for Ag deposited SBA-15 is 9.35 ± 2.06 nm (Figure 9). Nevertheless, the result for Ag deposited on SBA-15-4.2 is 10.98 ± 3.30 nm. Then, the actual loading content was detected by ICP-AES. There is a great decrease for the loading content of Ag/SBA-15-4.2 due to less surface area and small pore diameter. The loading content was about 2.53 wt% for Ag/SBA-15-4.2.

### Catalytic Activity

**FIGURE 10:**
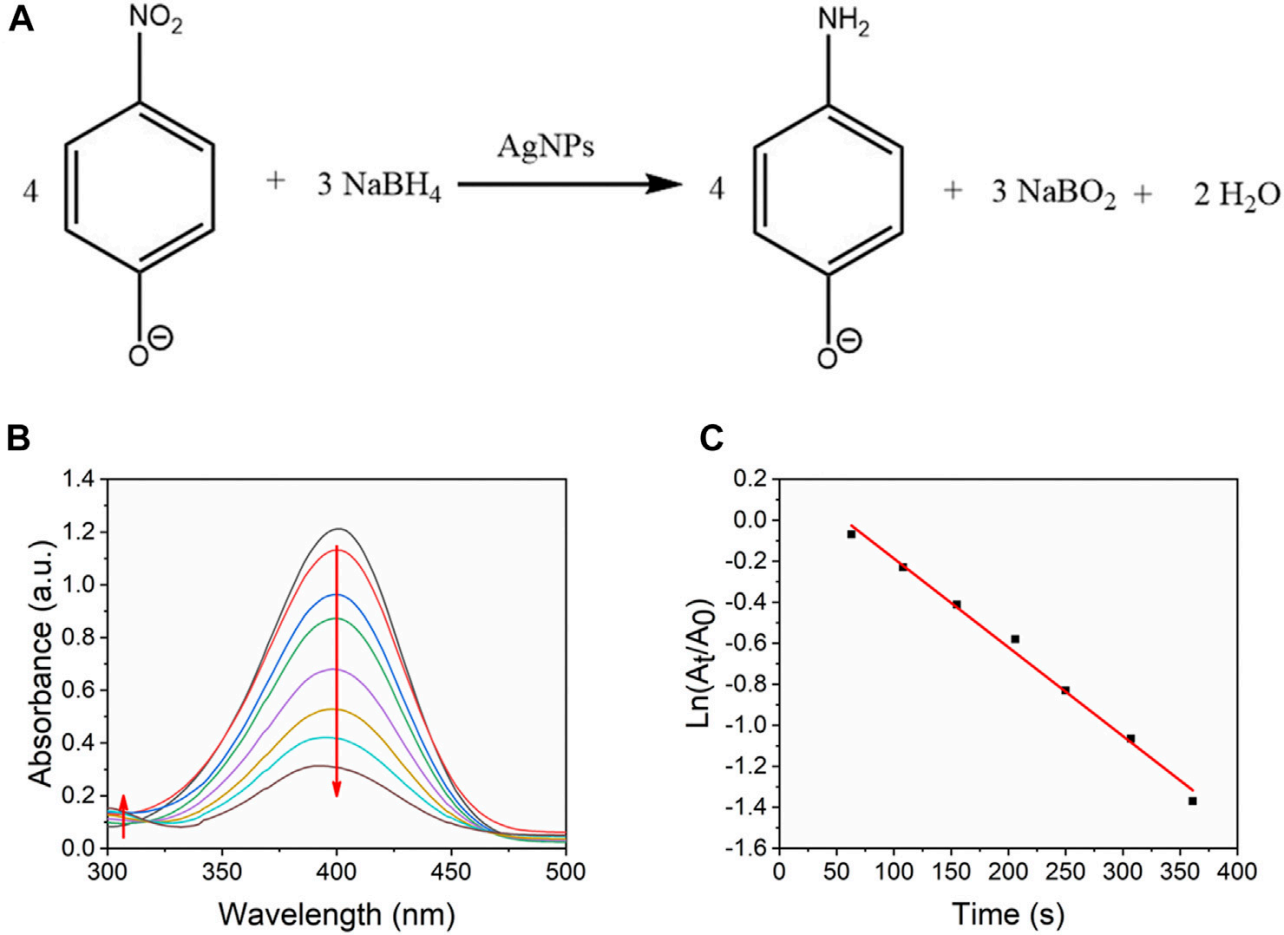
**(A)** Reduction of 4-NP to 4-AP by excess NaBH_4_. **(B)** UV-vis absorption spectra of reduction of 4-NP by Ag/SBA-15 synthesized at 17.5 s residence time as a function of time. **(C)** Kinetic analysis of the reaction.

Noble metal-based nanoparticles supported on mesoporous materials have shown excellent catalytic performances towards reduction of nitro-aromatic compounds and 4-nitrophenol is a harmful pollutant generated from dye, drugs and pesticides (Naik et al., 2011; Kumar et al., 2019; Manno et al., 2021). Also, the product 4-AP can be further utilized to synthesized drugs (Seo et al., 2017). Therefore, the catalytic activity could be evaluated by the fast reduction of 4-nitrophenol (4-NP) (Figure 10A). As shown in Figure 10B, in the absence of catalysts, 4-NP exhibit a stable absorbance peak at 400 nm. After adding the nanocatalyst, the 4-NP was reduced to 4-aminophenol (Jin et al., 2012). The adding amount of nanocatalysts was minimal, so the absorbance peak at 400 nm was almost determined by the concentration of 4-NP. The peak at 400 nm decreased with the reduction of 4-NP, and the peak around 300 nm increased because of the formation of 4-aminophenol.

The reduction of 4-NP was considered a pseudo-first-order reaction (Wu and Torrente-Murciano, 2018). Therefore, the rate of consumption of 4-nitrophenol was defined as follows:

$$r_{t}=\frac{dC_{A}}{dt}=K_{app}C_{A},$$ (1)

Where $C_{A}$ is the concentration of 4-nitrophenol, and $K_{app}$ is the apparent reaction rate constant in s^−1^.

According to Beer-Lambert law, the absorbance of a 4-NP solution is proportional to the 4-NP concentration. Then, a good linear equation, ln*A* vs time plot, was obtained:

$$-K_{app}t=ln\frac{A_{t}}{A_{0}}=ln\frac{C_{t}}{C_{0}}.$$ (2)

Here, $C_{0}$ is the initial concentration of the 4-NP before adding the catalysts and $C_{t}$ is the 4-NP concentration at a given time (t). $A_{t}$ and $A_{0}$ respectively represent the absorbance at a given time (t) and the initial concentration. A typical fitting for the detected results is shown in Figure 10C **.**

**FIGURE 11:**
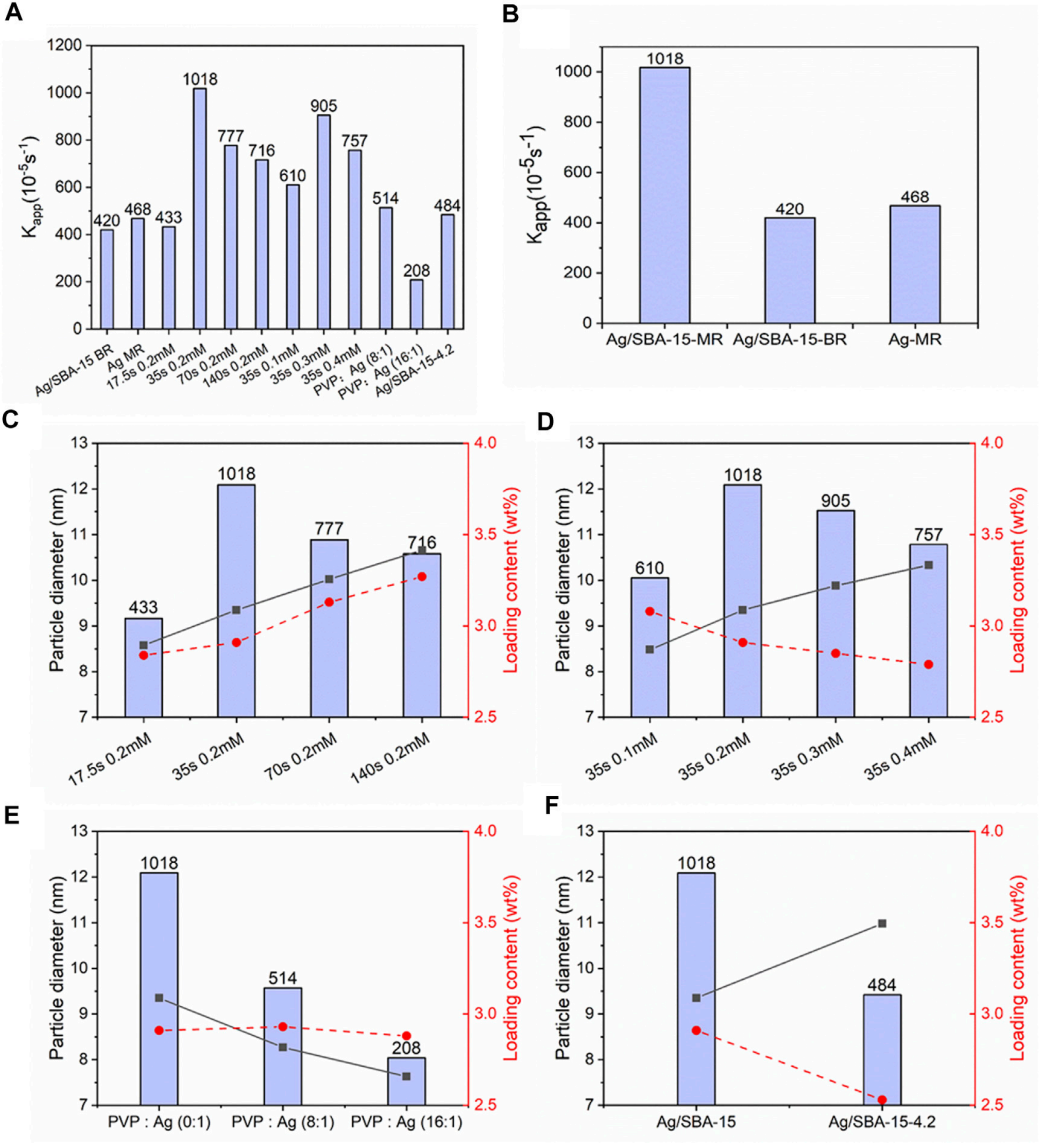
**(A)** Catalytic performance over all catalysts. **(B)** Catalysts synthesized at different condition. Ag/SBA-15-MR, Ag/SBA-15-BR, Ag-MR represents Ag/SBA-15 synthesized in the microreactor, Ag/SBA-15 synthesized in batch reactor and Ag synthesized in the microreactor, respectively. **(C)** Relationship between nanoparticles’ size dispersed on SBA-15, loading content and catalytic performance for Ag/SBA-15 synthesized at different flow rates. **(D)** Relationship between nanoparticles’ size dispersed on SBA-15, loading content and catalytic performance for Ag/SBA-15 synthesized with different initial concentrations. **(E)** Catalytic performance of Ag/SBA-15 synthesized with different concentration of PVP. **(F)** Catalytic activity of Ag/SBA-15 and Ag/BSA-15-4.2.

Catalytic performance of mesoporous silica nanocatalysts is related to many factors, such as size and size distribution of nanoparticles, actual loading contents and structure for support. Figure 11A shows the catalytic activity over all catalysts. The best catalytic performance was obtained when the supported nanoparticle was synthesized at 35 s residence time and initial concentration of 0.2 mM. The biggest apparent reaction rate constant is about 0.01018 s^−1^.

Seen from Figure 11B, Ag/SBA-15 synthesized in continuous flow has a better catalytic activity than that synthesized in conventional batch reactor due to the uniform nanoparticles on the support. Also, the introduction of support can enhance the dispersion and further enhance the activity, compared with the silver nanoparticle without SBA-15 synthesized in the microreactor.

In order to investigate the relationship between size, loading content and catalytic activity, Ag/SBA-15 at different condition were involved in testing the catalytic performance. It can be observed from Figures 11C,D that the catalytic performance does not increase with the decrease of size diameter or the increase of loading content. Actually, the rate of catalytic reaction should be higher at higher loading level due to more active sites (Naik et al., 2011). Nevertheless, in this process, according to the results in Figures 11C,D, compared with the effect of nanoparticles’ size, loading content does not mainly attribute to the change of catalytic activity when there is no big difference between the loading contents. Therefore, the morphology or size of silver nanoparticles could be the main factor for the activity difference (Li et al., 2014; Souza et al., 2014; Ran et al., 2015).

The effect of capping agents and support with different specific surface area have been evaluated as showed in Figures 11E,F. Figure 11E indicates an appreciable decrease of catalytic reaction rate with the increase of PVP’s concentration, mainly due to PVP’s residuals resistant to removal (Niu and Li, 2014). During the synthesis of nanoparticles, capping agents can encapsulate on the surface of the nanoparticles, which inhibit the agglomeration. On the other hand, capping agents act as a barrier to hinder the access of reactants to active sites (Li et al., 2012; Shao et al., 2013). So, when increasing the PVP’s concentration, more unremoved PVP will further inhibit the activity. Catalytic activity of supports with different surface area was investigated as seen from Figure 11F. When silver nanoparticles are deposited on SBA-15-4.2, which has a smaller surface area than SBA-15, the catalytic reaction rate decreases as a result of decrease of loading content and bigger nanoparticles’ size (Hashimi et al., 2019).

## Conclusion

In summary, uniform Ag/SBA-15 (with Ag nanoparticles of 7–11 nm in diameter) has been synthesized in a continuous flow reactor. The flow rate and concentration change has less effect on the supported nanocatalysts for both size and loading content. Remarkably, the introduction of support and capping agents can effectively disperse the nanoparticles and inhibit aggregation. The increase of concentration of capping agents and surface area of mesoporous support can further enhance the effect, thus synthesizing smaller uniform nanoparticles for supported nanocatalysts. Furthermore, the relationship between catalytic performance, loading content and size has been evaluated, and the best catalytic activity is achieved for Ag/SBA-15 synthesized at 35 s residence time and 0.2 mM initial concentration, mainly due to the effect of size or morphology of silver nanoparticles. This work provides a method for continuous synthesis of uniformly dispersed mesoporous SBA-15 nanocatalysts. In addition, it can help further understanding of the size and catalytic performance of nanomaterials.

## Data Availability

The original contributions presented in the study are included in the article/Supplementary Material, further inquiries can be directed to the corresponding authors.
